# What is the correct therapeutic approach in patients with advanced HIV infection associated with ≥ 3 AIDS defining illness?

**DOI:** 10.1186/1471-2334-13-S1-P22

**Published:** 2013-12-16

**Authors:** Alina Cozma, Daniela Camburu, Georgeta Ducu, Mihaela Ionică, Manuela Podani, Roxana Dumitriu, Șerban Benea, Cozmina Andrei, Monica Zăstran, Mădălina Simoiu, Otilia Elisabeta Benea

**Affiliations:** 1 National Institute for Infectious Diseases “Prof. Dr. Matei Balş”, Bucharest, Romania; 2 Carol Davila University of Medicine and Pharmacy, Bucharest, Romania

## Background

Specific treatment of opportunistic infections associated with HIV infection raises real difficulties in current practice due to side effects, drug interactions and immune reconstitution syndrome. For advanced immunosuppression initiation of the antiretroviral therapy may be a therapeutic emergency.

## Case report

We present a case of a 29 years old patient, MSM, confirmed with HIV infection in 2007 but who do not accept the diagnosis and returns to our service in 2013. He was diagnosed with generalized Kaposi sarcoma (extensive skin lesions, sores in the mouth and cavum, lung injury; the clinical diagnosis was confirmed by skin biopsy and lung CT), cerebral toxoplasmosis (specific to brain MRI image with anti-toxoplasma IgG positive), disseminated tuberculosis (positive blood cultures for *M tuberculosis*) and genital herpes with large lesions and necrotic component, at a CD4 = 2 cells/cmm. Treatment was complex, for getting every opportunistic infection; because of the complexity of the case and the hematologic associated events (severe leucopenia, anemia and thrombocytopenia) has not been discussed the specific therapy for Kaposi' sarcoma. After 6 weeks of antinfectious treatment and correction of associated metabolic and hematological disorders, the antiretroviral therapy with Abacavir, Epivir and Raltegravir was introduced. Without developing immune reconstitution syndrome the evolution was unfavorable to death.

## Conclusion

We discuss the correct therapeutic approach for the treatment of AIDS-defining diseases associated and the proper management of this type of patient.

